# Knowledge, attitude, and practice toward interstitial lung disease among patients: a cross-sectional study

**DOI:** 10.3389/fmed.2024.1397659

**Published:** 2024-06-20

**Authors:** Wenjie Xu, Qiuhong Li, Yanjun Mao, Yan He

**Affiliations:** ^1^Department of Respiratory and Critical Care Medicine No. 1, Shanghai Pulmonary Hospital, Shanghai, China; ^2^Nursing Department, Shanghai Pulmonary Hospital, Shanghai, China; ^3^Department of Respiratory and Critical Care Medicine No. 2, Shanghai Pulmonary Hospital, Shanghai, China

**Keywords:** interstitial lung diseases, surveys and questionnaires, cross-sectional study, knowledge, attitude, practice

## Abstract

**Objective:**

To explore the knowledge, attitude, and practice (KAP) toward interstitial lung disease (ILD) among patients and analyze the factors affecting KAP.

**Methods:**

This cross-sectional study enrolled patients with ILD treated at the Respiratory Department of Shanghai Pulmonary Hospital between January 2023 and June 2023. A self-administered questionnaire was developed to evaluate their KAP toward ILD through convenient sampling. Multivariate regression analysis and structural equation model (SEM) were used to analyze the factors influencing KAP and their interactions.

**Results:**

A total of 397 patients were enrolled, with 61.71% male. The mean KAP scores were 4.60 ± 3.10 (possible range: 0–12), 16.97 ± 2.16 (possible range: 5–25), and 32.60 ± 7.16 (possible range: 9–45), respectively. Multivariate logistic regression analysis showed that junior high school [OR = 2.003, 95%CI: 1.056–3.798, *p* = 0.033], high school and above [OR = 2.629, 95%CI: 1.315–5.258, *p* = 0.006], and duration of disease ≥5 years [OR = 1.857, 95%CI: 1.132–3.046, *p* = 0.014] were independently associated with adequate knowledge. The knowledge [OR = 1.108, 95%CI: 1.032–1.189, *p* = 0.005] and duration of disease ≥5 years [OR = 0.525, 95%CI: 0.317–0.869, *p* = 0.012] were independently associated with a positive attitude. The knowledge [OR = 1.116, 95%CI: 1.036–1.202, *p* = 0.004], attitude [OR = 1.180, 95%CI: 1.061–1.312, *p* = 0.002], and the age of >70 years [OR = 0.447, 95%CI: 0.245–0.817, *p* = 0.009] were independently associated with the proactive practice. SEM showed that patients’ knowledge of ILD directly affected their attitude (β = 0.842, *p* < 0.001) and practice (β = 0.363, *p* < 0.001), and their attitude also affected their practice (β = 0.347, *p* = 0.014).

**Conclusion:**

Patients with ILD in China had poor knowledge, intermediate attitude, and proactive practice toward ILD, which suggests that the health education of patients should be further strengthened.

## Introduction

Interstitial lung disease (ILD) refers to the lung disorders characterized by inflammation and fibrosis of the interstitium (1). There are more than 200 subtypes of ILD, including idiopathic pulmonary fibrosis (IPF), sarcoidosis, hypersensitivity pneumonitis, and connective tissue disease-associated ILD (2). ILD impairs the lungs’ ability to function properly, and leads to symptoms such as breathlessness, coughing, and reduced exercise tolerance (3). From 1990 to 2019, the global incidence and mortality rates due to ILD have significantly increased from 118.6 to 166.63%, respectively (4). From 2005 to 2020, the prevalence of ILD rose from 24.7 to 33.6 per 100,000 population, with an average annual percent change of 1.94% (5). Patients with ILD often experience a significant decline in their quality of life due to symptoms such as dyspnea, cough, and fatigue, which can impair their ability to perform daily activities and lead to social isolation (6, 7). Moreover, ILD can adversely affect physical function by reducing exercise capacity and lung function, resulting in increased morbidity and mortality (8). Given the considerable burden of ILD on affected individuals and healthcare systems, understanding patients’ knowledge, attitude and practice (KAP) towards ILD is essential for future prevention and management.

The KAP study assesses individuals’ understanding, perceptions, and behaviors related to a specific health issue through structured questionnaires (9). Understanding and adhering to diagnostic practices, such as pulmonary function tests, high-resolution computed tomography scans, and bronchoscopy, can help confirm the diagnosis of ILD and assess disease severity (10, 11). Positive attitude towards lifestyle modifications, such as smoking cessation and avoidance of environmental pollutants, can reduce the risk of respiratory infections, which further prevents the occurrence of ILD symptoms (12). Besides, active compliance to pharmacological interventions, including corticosteroids, immunosuppressive agents, and antifibrotic medications, may mitigate inflammation and fibrosis in the lungs (13). Published studies indicated that while ILD patients have demonstrated favorable attitudes toward their condition and its management, their knowledge, particularly concerning disease exacerbations and progression, has been found to be inadequate (14–16). However, prior researches were mainly on IPF, and limited attention has been paid regarding the KAP status among patients with different ILD subtypes (17–19). Additionally, the impact of socio-demographic factors on patients’ KAP remains unclear. Considering the importance of enhancing ILD prevention and management, a thorough comprehension of patients’ KAP and its determining factors is imperative.

This study aimed to address the research gap by conducting a cross-sectional investigation into the KAP of patients towards ILD. Besides, the influential factors of KAP were explored, which had implications for developing education interventions among targeted populations. By elucidating the factors influencing patients’ behaviors and practices related to ILD management, the study seeks to provide valuable insights into patient-centered care and the development of support programs to improve clinical outcomes for individuals living with ILD.

## Methods

### Study design and participants

Patients with ILD treated at the Respiratory Department of Shanghai Pulmonary Hospital between January 2023 and June 2023 were enrolled in this cross-sectional study. The convenient sampling method was used. The inclusion criteria were: (1) inpatients with a confirmed diagnosis of ILD; (2) ≥ 18 years old; (3) with good enough reading and writing skills in the Chinese language to complete the questionnaire independently. The diagnostic criteria for ILD involves clinical, radiological, and pathological evaluations (20). Clinically, patients often present with symptoms such as chronic dry cough, progressive dyspnea, and fatigue. Radiological assessment typically includes high-resolution computed tomography (HRCT) scans, which can reveal patterns like ground-glass opacities, reticulation, and honeycombing indicative of ILD. Pulmonary function tests (PFTs) can show the restrictive patterns with reduced lung volumes and impaired gas exchange. The exclusion criteria were: (1) patients with mental illness or consciousness disorders and (2) patients with interstitial pneumonia who required ventilator support.

This study was approved by the Ethics Committee of Shanghai Pulmonary Hospital (approval number: K22-001), and all participants provided informed consent.

### Sample size calculation

The calculation of sample size was based on the following formula employed in the cross-sectional study (21):


$$n={\left(\frac{Z_{1-\frac{\alpha }{2}}}{\delta }\right)}^{2}\times p\times \left(1-p\right)$$

where n denoted the sample size. Besides, *p* value was assumed to be 0.5 to achieve the maximum sample size. a refers to the type I error, which was set to 0.05 in this case. Subsequently, $Z_{1-\frac{\alpha }{2}}$ was yielded 1.96. δ represents the effect sizes between groups, which was determined as 0.06, and at least 267 participants should be required. As regards the 20% of non-response rate, a total of 334 participants are required to be involved.

### Questionnaire design

A self-administered questionnaire was designed referring to previous studies (22–24), and was reviewed by three respiratory epidemiologists, five specialists in ILD, and three specialists in Nursing. A pre-test (n = 30) was conducted, generating Cronbach’s α of 0.824, which was indicative of good internal consistency of the questionnaire. The Content Validity Ratio (CVR) was 0.80, indicating a high level of expert agreement on the essentiality of the questionnaire items. The Content Validity Index (CVI) was calculated to be 0.82, demonstrating good content validity for the questionnaire.

The final questionnaire included 35 items across four following dimensions: (1) the demographic characteristics, which consisted of 9 items that covered the gender, age, education, place of residence, type of ILD (The type of ILD were self-reported, not clinical diagnosed), duration of disease, smoking, family history of ILD, and experience of treatment for ILD; (2) the knowledge dimension (12 items); (3) the attitude dimension (5 items); (4) the practice dimension (9 items). Items in the knowledge dimension were scored with 1 point for each correct answer and 0 points for incorrect or unclear answers, with a final score ranging from 0–12 points. The attitude and practice dimensions were evaluated on a five-point Likert scale, where answers were scored from “strongly agree” (5 points) to “strongly disagree” (1 point) in the attitude dimension and from “always” (5 points) to “never” (1 point) in the practice dimension. The total score range was 5–25 points in the attitude dimension and 9–45 points in the practice dimension. For all three dimensions, >70% of the total KAP scores represented “adequate knowledge,” “positive attitude,” and “proactive practice” (25).

### Data collection

The paper questionnaires were distributed to inpatients with ILD through the convenient sampling method. A unified training was conducted for members of the research team prior to the survey. Before the survey, the researchers explained the purpose of the study to each participant, and all participants signed the informed consent. During the survey, the researchers were at the participants’ disposal, explaining any unclear questionnaire descriptions. All data were anonymous. Members of the research team checked all questionnaires for completeness, internal coherence, and reasonableness after questionnaire collection. Questionnaires with logical errors or consistent answers to all questions were evaluated as invalid and excluded.

### Statistical analysis

SPSS 22.0 (IBM Corp., Armonk, NY, United States) was used for this analysis. Continuous variables with normal distribution were presented as Mean ± Standard Deviation (SD), and Student’s t-test or One-way Analysis of Variance (ANOVA) was performed to compare continuous variables between two groups or among multiple groups. Categorical variables were presented as numbers (percentages). Pearson’s correlation analysis was used to analyze the correlation between the patients’ KAP towards ILD. Multivariate logistic regression analysis was used to explore the factors associated with KAP; variables with *p* < 0.1 in the univariate analysis were included in the multivariate analysis. The structural equation model (SEM) was constructed, and the hypothesis was as follows: (1) patients’ knowledge of ILD directly affects their attitudes towards ILD; (2) patients’ attitudes towards ILD directly affect their practice towards ILD; and (3) patients’ knowledge towards ILD directly affects their practice towards ILD. Comparative Fit Index (CFI), Goodness of Fit Index (GFI), Adjusted goodness of fit index (AGFI), and Root Mean Square Error of Approximation (RMSEA) were used to evaluate the model fit of SEM. A two-sided *p* < 0.05 represented statistical significance.

## Results

Among the 397 participants, 245 (61.71%) were male, and 165 (41.56%) were aged between 60 and 70 years. The majority (74.56%) had an ILD duration of less than 5 years, and 26.95% had not undergone treatment for ILD. Idiopathic pulmonary fibrosis was the most prevalent ILD subtype (15.37%), followed by other types (9.82%) and acute interstitial pneumonia (8.56%). Additionally, 59.70% of participants were unaware of their specific ILD subtype. Patients’ knowledge, attitude, and practice scores of ILD were 4.60 ± 3.10, 16.97 ± 2.16, and 32.60 ± 7.16, respectively. Higher knowledge scores were observed in patients from urban areas (*p* = 0.009), with the duration of disease ≥5 years (*p* < 0.001), and those treated for ILD (p < 0.001). There were also significant differences in the knowledge and attitude scores in patients with different education and types of ILD (all *p* < 0.05). Moreover, a significant difference was found in the practice scores among patients with different smoking status (*p* = 0.005) (Table 1).

**Table 1:** Baseline characteristics and KAP of the study population towards ILD.

Variables	N (%)	Knowledge scores		Attitude scores		Practice scores	
		Mean ± SD	*p*	Mean ± SD	*p*	Mean ± SD	*p*
Total scores	397	4.60 ± 3.10		16.97 ± 2.16		32.60 ± 7.16	
Gender			0.079		0.970		0.171
Male	245 (61.71)	4.81 ± 3.01		16.98 ± 2.10		32.22 ± 7.18	
Female	152 (38.29)	4.25 ± 3.21		16.97 ± 2.27		33.23 ± 7.11	
Age (years)			0.503		0.398		0.051
<60	125 (31.49)	4.39 ± 3.09		16.96 ± 2.31		33.66 ± 7.52	
60–70	165 (41.56)	4.81 ± 3.10		17.12 ± 2.14		32.61 ± 6.94	
>70	107 (26.95)	4.51 ± 3.11		16.76 ± 2.01		31.36 ± 6.93	
Education			<0.001		0.010		0.362
Primary school and below	100 (25.19)	3.58 ± 3.12		16.84 ± 1.86		31.99 ± 7.06	
Junior high school	172 (43.32)	4.74 ± 3.02		16.70 ± 2.18		32.45 ± 6.52	
High school and above	125 (31.49)	5.21 ± 3.00		17.45 ± 2.29		33.31 ± 8.03	
Place of residence			0.009		0.226		0.560
Rural area	129 (32.49)	4.02 ± 3.09		16.78 ± 1.75		32.30 ± 6.83	
Urban area	268 (67.51)	4.88 ± 3.07		17.06 ± 2.33		32.75 ± 7.32	
Type of ILD			<0.001		0.001		0.289
Idiopathic pulmonary fibrosis (IPF)	61 (15.37)	5.84 ± 2.82		17.62 ± 2.25		32.11 ± 6.15	
Acute interstitial pneumonia (AIP)	34 (8.56)	4.26 ± 2.62		16.35 ± 2.16		30.59 ± 7.14	
Diffuse panbronchitis (DPB)	7 (1.76)	5.43 ± 3.41		17.14 ± 2.04		33.14 ± 5.84	
Pulmonary alveolar proteinosis (PAP)	19 (4.79)	6.16 ± 2.52		18.26 ± 1.88		34.53 ± 5.82	
Others	39 (9.82)	5.15 ± 3.39		16.13 ± 2.00		31.49 ± 6.70	
Do not know	237 (59.70)	4.08 ± 3.09		16.92 ± 2.11		33.03 ± 7.57	
Duration of disease			<0.001		0.077		0.228
<5 years	296 (74.56)	4.23 ± 3.02		17.08 ± 2.20		32.86 ± 7.02	
≥5 years	101 (25.44)	5.68 ± 3.09		16.64 ± 2.00		31.86 ± 7.55	
Smoking			0.139		0.488		0.005
Never smoking	195 (49.12)	4.43 ± 3.19		17.01 ± 2.23		33.23 ± 6.86	
Have quit smoking	174 (43.83)	4.91 ± 3.00		17.01 ± 2.11		32.56 ± 7.09	
Still smoking	28 (7.05)	3.86 ± 2.90		16.50 ± 1.97		28.54 ± 8.45	
Family history of ILD			0.168		0.742		0.764
Yes	35 (8.82)	5.29 ± 3.23		16.86 ± 1.97		32.26 ± 7.08	
No	362 (91.18)	4.53 ± 3.08		16.98 ± 2.18		32.64 ± 7.18	
Experience of treatment for ILD			0.003		0.836		0.904
Yes	290 (73.05)	4.88 ± 3.04		16.96 ± 2.07		32.63 ± 6.82	
No	107 (26.95)	3.83 ± 3.13		17.01 ± 2.40		32.53 ± 8.04	

The detailed distribution of patients’ KAP of ILD is shown in Supplementary Tables S1–S3. Only 43 (10.83%) patients knew that ILD is more than pulmonary fibrosis, 84 (21.16%) were familiar with the typical signs of ILD, and 91 (22.92%) provided correct answers on the reversibility of ILD (Supplementary Table S1). Among the ILD patients, 38 (9.57%) and 110 (27.71%) individuals expressed strong agreement or agreement that their dyspnea or tachypnea symptoms were likely caused by obesity or aging rather than ILD. Similarly, 130 patients (32.75%) strongly concurred and 197 (49.62%) concurred with the importance of seeking medical examinations when experiencing symptoms. Additionally, 99 patients (24.94%) strongly concurred and 183 patients (46.10%) concurred that ILD significantly impacted their daily lives. Furthermore, 78 patients (19.65%) strongly concurred and 204 (51.39%) concurred that their ILD symptoms would be considerably alleviated with treatments, while 89 patients (22.42%) strongly agreed and 193 (48.61%) agreed on the necessity of participating in educational courses to enhance their understanding of ILD (Supplementary Table S2). As for practice, 245 patients (61.71%) reported that they always followed/would always follow the doctor’s instructions for medication strictly; however, merely 60 patients (15.11%) claimed that they followed/would follow the doctor’s instructions for pulmonary rehabilitation exercises (Supplementary Table S3).

Pearson correlation analysis revealed a positive correlation between the knowledge scores of patients, their attitude scores (r = 0.169, *p* = 0.001), and practice scores (r = 0.242, *p* < 0.001). In addition, the attitude scores were also positively correlated with the practice scores (r = 0.225, p < 0.001) (Table 2). Multivariate regression analysis showed that high school education and above [OR = 2.444, 95%CI: 1.206–4.955, *p* = 0.013], unknown with the type of ILD [OR = 0.509, 95%CI: 0.276–0.941, *p* = 0.031], and duration of disease ≥5 years [OR = 1.857, 95%CI: 1.108–3.112, *p* = 0.019] were independently associated with adequate knowledge. The knowledge scores [OR = 1.087, 95%CI: 1.010–1.170, *p* = 0.026], acute interstitial pneumonia [OR = 0.268, 95%CI: 0.102–0.703, *p* = 0.007], other type of ILD [OR = 0.254, 95%CI: 0.101–0.637, *p* = 0.003], and duration of disease ≥5 years [OR = 0.550, 95%CI: 0.317–0.869, *p* = 0.020] were independently associated with positive attitude. Furthermore, patients’ knowledge scores [OR = 1.116, 95%CI: 1.036–1.202, *p* = 0.004], attitude scores [OR = 1.180, 95%CI: 1.061–1.312, *p* = 0.002], and the age of >70 years [OR = 0.447, 95%CI: 0.245–0.817, *p* = 0.009] were independently associated with proactive practice (Table 3).

**Table 2:** Correlation analysis of KAP towards ILD.

	Knowledge	Attitude	Practice
Knowledge	1		
Attitude	0.169 (*p* = 0.001)	1	
Practice	0.242 (*p* < 0.001)	0.225 (*p* < 0.001)	1

**Table 3:** Multivariate analysis of KAP.

Variables	Multivariate analysis of knowledge		Multivariate analysis of attitude		Multivariate analysis of practice	
	OR (95%CI)	*p*	OR (95%CI)	*p*	OR (95%CI)	*p*
Knowledge	/	/	1.087 (1.010–1.170)	0.026	1.116 (1.036–1.202)	0.004
Attitude	/	/	/	/	1.180 (1.061–1.312)	0.002
Age, years						
<60					Reference	
60–70					0.621 (0.372–1.036)	0.068
>70					0.447 (0.245–0.817)	0.009
*Education*						
Primary school and below	Reference		Reference			
Junior high school	1.909 (0.997–3.656)	0.051	1.107 (0.621–1.974)	0.731		
High school and above	2.444 (1.206–4.955)	0.013	1.669 (0.878–3.175)	0.118		
*Place of residence*						
Rural area	Reference		Reference			
Urban area	1.200 (0.689–2.089)	0.520	1.356 (0.809–2.272)	0.248		
*Type of ILD*						
Idiopathic pulmonary fibrosis (IPF)	Reference					
Acute interstitial pneumonia (AIP)	0.546 (0.214–1.396)	0.207	0.268 (0.102–0.703)	0.007		
Diffuse panbronchitis (DPB)	1.094 (0.207–5.796)	0.916	1.057 (0.210–5.335)	0.946		
Pulmonary alveolar proteinosis (PAP)	2.063 (0.702–6.066)	0.188	1.235 (0.409–3.726)	0.708		
Others	0.910 (0.392–2.112)	0.826	0.254 (0.101–0.637)	0.003		
Do not know	0.509 (0.276–0.941)	0.031	0.550 (0.302–1.003)	0.051		
*Duration of disease*						
<5 years	Reference		Reference			
≥5 years	1.857 (1.108–3.112)	0.019	0.531 (0.312–0.906)	0.020		
Experience of treatment for ILD						
Yes	1.301 (0.754–2.244)	0.345				
No	Reference					

**Figure 1:**
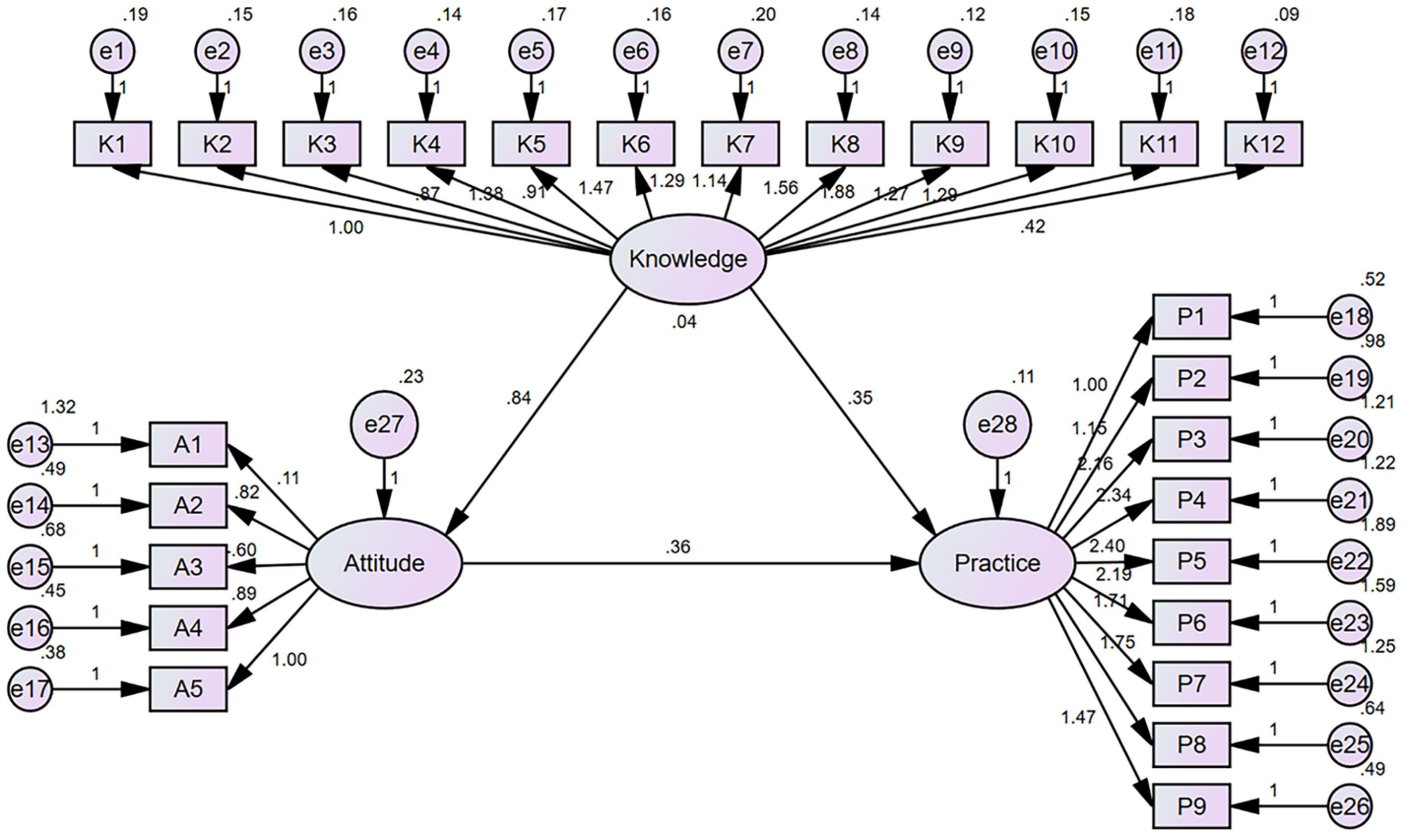
Structural equation model of patients’ KAP.

Except for the CFI of 0.782, the fitting index of the SEM (CMIN/DF = 2.653, RMSEA = 0.065, AGFI = 0.843, GFI = 0.868) showed that the model was acceptable (Table 4). SEM results showed that patients’ knowledge of ILD directly affected their attitudes (β = 0.842, *p* < 0.001) and practices towards ILD (β = 0.363, p < 0.001), and the attitudes of patients towards ILD also directly and positively affected their practices towards ILD (β = 0.347, *p* = 0.014) (Table 5; Figure 1).

**Table 4:** Fitting indicators of the SEM model.

Indicators	Reference standard	Measured value
CMIN/DF	1–3 excellent	2.653
RMSEA	<0.08 good	0.065
AGFI	>0.8 good	0.843
CFI	>0.8 good	0.782
GFI	>0.8 good	0.868

**Table 5:** Path relationships of KAP in SEM.

Path relationships			Estimate	*p*
Attitude	<−--	Knowledge	0.842	<0.001
Practice	<−--	Attitude	0.363	<0.001
Practice	<−--	Knowledge	0.347	0.014

## Discussion

The present study revealed that the ILD patients had poor knowledge, intermediate attitude, and proactive practices toward ILD. Our results showed the existing KAP gaps among ILD patients, emphasizing the importance of promoting patient education in the ILD areas. The present study highlights the need for interventions and educational programs to address the knowledge gap, enhance patients’ attitudes toward ILD, and promote more proactive practices in disease management among patients with ILD in China.

Unlike previous surveys that primarily focused on the KAP of specialists and patients with IPF (26–28), this study examined ILD-related KAP among ILD patients with different subtypes, providing valuable data for understanding the KAP status among patients of various ILD subtypes. The results revealed a lack of awareness about their specific ILD subtype in most patients (59.70%). Moreover, only a minority (22.92%) had an accurate understanding of ILD reversibility, indicating low disease awareness. These findings aligned with earlier research involving patients with connective tissue disease-associated ILD and IPF, where patients often struggled to comprehend their diagnosis and prognosis (14, 29). Besides, only 10.83% possessed accurate knowledge about ILD encompassing conditions beyond just pulmonary fibrosis, which could impede appropriate management. Moreover, only 21.16% of the patients were familiar with the typical signs of ILD, which is somewhat concerning. Recognizing symptoms constitutes the initial step in seeking medical assistance, hence a lack of awareness might exacerbate the condition and disrupt the prognosis of ILD. Therefore, efforts involving healthcare providers, patient advocacy groups, and public health initiatives are necessary to educate the population about the diverse nature of ILD and its common signs.

In the attitude dimension, 37.28% of participants attributed their dyspnea or tachypnea symptoms more to obesity or aging than ILD, which reflected the broader awareness of the impacts of these prevalent health factors on respiratory issues (30, 31). However, due to dyspnea and tachypnea as hallmark symptoms of ILD, the delay in awareness could impact disease management (32). Therefore, targeted educational interventions to enhance public understanding of respiratory symptoms and their diverse etiologies are warranted. Similarly, the majority (71.04%) strongly agreed that ILD significantly disrupted their daily lives. ILD imposes systemic inflammation, dyspnea, chronic cough, and fatigue and affects daily life through anxiety, depression, and frustration (22, 33). This emphasizes the need for a multidisciplinary approach to address ILD’s physical and psychosocial implications. In addition, a considerable number of participants (71.04%) acknowledged the alleviation of ILD symptoms following appropriate treatments. This optimism might encourage patients to adhere to treatment plans, reinforcing the importance of effective patient-provider communication and realistic treatment expectations. Furthermore, recognizing the need for educational courses to enhance the understanding of ILD indicated a patients’ willingness to actively engage in their healthcare. This presents an opportunity for healthcare providers to develop and implement educational programs tailored to ILD patients, empowering them to manage their condition more effectively.

High adherence to prescribed medication (61.71%) suggested a generally positive approach to the pharmaceutical management of ILD. This is a crucial aspect in managing ILD, as medications help with symptom control, disease progression management, and overall improvement in the quality of life (1). Conversely, the lowest percentage (15.11%) of patients who strictly adhered to or expressed willingness to adhere to pulmonary rehabilitation exercises was concerning. Pulmonary rehabilitation is an essential component of ILD management encompassing a range of therapies, exercises, and educational interventions designed to optimize patients’ physical and social well-being (8). Several factors could account for these findings, including the lack of awareness of the benefits and perceived burden or discomfort associated with pulmonary rehabilitation exercises. Addressing this gap requires a multi-faceted approach involving increased patient education, individualized exercise plans, and psychological support.

The influential factors of KAP scores were further identified using multivariate analysis. First, education level was positively associated with knowledge score. Higher education levels often correlate with better health literacy, enabling individuals to comprehend complex medical information and engage effectively in their healthcare (34). Second, having an unknown ILD subtype was negatively associated with the knowledge scores. Patients with clear diagnoses might receive more targeted information and education about their condition. Therefore, providing detailed information about ILD subtypes could reduce this knowledge gap. Third, the disease duration of ≥5 years was associated with higher levels of knowledge. Long-term experience managing the disease, interactions with healthcare professionals, and self-education could contribute to higher knowledge. Fourth, the negative association between certain ILD subtypes (acute interstitial pneumonia and other types) and attitude scores warranted careful consideration. These findings suggested that patients with these subtypes might have more severer symptoms or prognosis, thereby reducing their attitudes scores. Lastly, the negative association between age (>70 years) and practice emphasized the need for targeted interventions among older populations. Older individuals might face barriers such as physical limitations, cognitive challenges, or lack of access to resources, which could hinder proactive engagement in managing their ILD (35).

The SEM outcomes elucidated direct and positive associations between the knowledge and attitude of ILD patients and their practical actions, highlighting the significance of augmenting patients’ knowledge and bolstering their attitude to foster engagement in ILD management. These insights could potentially guide the development of KAP-based educational initiatives and interventions for ILD patients in the future. Additionally, healthcare providers can apply our findings to enhance the quality of long-term ILD management by prioritizing patient education within clinical practice.

The present study has several limitations. First, the sample size was relatively small, which could affect the findings’ statistical power and generalizability. Future multicenter, nationwide studies with a larger sample size are needed to confirm this data. Second, the convenience sampling method that was used to distribute questionnaires might introduce selection bias. Consequently, these findings should be extrapolated with caution to other ILD subtypes or broader populations. Lastly, there is the possibility of self-reporting bias, where participants might provide responses influenced by social desirability or subjective interpretations (36).

## Conclusion

ILD patients in China had poor knowledge, intermediate attitudes, and proactive practice towards the condition. Healthcare providers should offer educational courses, particularly for newly diagnosed, less educated, and elderly individuals, to enhance their understanding of ILD. This approach can foster positive attitudes and encourage proactive engagement in ILD-related practices.

## Data availability statement

The original contributions presented in the study are included in the article/Supplementary material, further inquiries can be directed to the corresponding authors.

## Ethics statement

The studies involving humans were approved by this study was approved by the Ethics Committee of Shanghai Pulmonary Hospital (approval number: K22-001), and all participants provided informed consent. The studies were conducted in accordance with the local legislation and institutional requirements. The participants provided their written informed consent to participate in this study.

## Author contributions

WX: Investigation, Resources, Supervision, Validation, Writing – original draft, Writing – review & editing. QL: Data curation, Project administration, Software, Visualization, Writing – original draft, Writing – review & editing. YM: Funding acquisition, Methodology, Software, Validation, Writing – original draft, Writing – review & editing. YH: Methodology, Project administration, Resources, Software, Writing – original draft, Writing – review & editing.
