# Supplementary material for: Knowledge, attitude, and practice toward interstitial lung disease among patients: a cross-sectional study
Source: Front Med (Lausanne). 2024 Jun 20;11:1397659. doi: 10.3389/fmed.2024.1397659 (PMC11222668; doi:10.3389/fmed.2024.1397659)
Supplement: Supplementary file 1 [file Table_1.DOCX]

**Table S1.** Distributions of patients’ knowledge towards ILD

| **Knowledge** | **Correct (n, %)** | **Scores** |
| --- | --- | --- |
| **1. Interstitial lung disease (ILD) is usually not malignant and is not caused by infectious pathogens.** | 135(34.01) | 0.34±0.47 |
| **2. ILD is a reversible chronic disease.** | 91(22.92) | 0.23±0.42 |
| **3. New infections can aggravate ILD.** | 248(62.47) | 0.62±0.48 |
| **4. Finger clubbing is the most typical sign of ILD.** | 84(21.16) | 0.21±0.41 |
| **5. ILD may be related to the environment, occupation and drugs.** | 175(44.08) | 0.44±0.50 |
| **6. ILD is categorized into acute, chronic and subacute types.** | 129(32.49) | 0.32±0.47 |
| **7. Active tachypnea is often the first symptom of ILD to visit the hospital.** | 228(57.43) | 0.57±0.50 |
| **8. Antibiotics and glucocorticoids are commonly used in the acute exacerbation of ILD.** | 140(35.26) | 0.35±0.48 |
| **9. Treatment for ILD can delay the development of pulmonary fibrosis.** | 200(50.38) | 0.50±0.50 |
| **10. Bronchoscopy is a commonly used pathological examination for ILD.** | 123(30.98) | 0.31±0.46 |
| **11. ILD is contagious.** | 229(57.68) | 0.58±0.49 |
| **12. ILD is none other than pulmonary fibrosis.** | 43(10.83) | 0.11±0.31 |

**Table S2.** Distributions of patients’ attitudes towards ILD

|  | **Strongly agree** **(n,%)** | **Agree (n,%)** | **Neutrality (n,%)** | **Disagree (n,%)** | **Strongly disagree (n,%)** |
| --- | --- | --- | --- | --- | --- |
| **1. When I have dyspnea or tachypnea, I do not think of it as ILD. The symptoms are more likely to be caused by my obesity or old age.** | 38(9.57) | 110(27.71) | 99(24.94) | 12(28.21) | 38(9.57) |
| **2. I think it is necessary to go to the hospital for examinations when I have symptoms such as dyspnea, dry cough, etc.** | 130(32.75) | 197(49.62) | 52(13.10) | 16(4.03) | 2(0.50) |
| **3. I think ILD has seriously affected my life.** | 99(24.94) | 183(46.10) | 88(22.17) | 24(6.05) | 3(0.76) |
| **4. I believe my ILD symptoms will be much relieved after treatments.** | 78(19.65) | 204(51.39) | 98(24.69) | 13(3.27) | 4(1.01) |
| **5. I think it is necessary to attend education courses to get more knowledge about ILD.** | 89(22.42) | 193(48.61) | 103(25.94) | 9(2.27) | 3(0.76) |

**Table S3.** Distributions of patients’ practices towards ILD

|  | **Always (n, %)** | **Usually (n, %)** | **Sometimes (n, %)** | **Occasionally (n, %)** | **Never (n, %)** |
| --- | --- | --- | --- | --- | --- |
| **1. I followed/would follow the doctor's instructions for medication strictly.** | 245(61.71) | 104(26.20) | 37(9.32) | 6(1.51) | 5(1.26) |
| **2. I kept/would get enough sleep during illness.** | 104(26.20) | 135(34.01) | 96(24.18) | 49(12.34) | 13(3.27) |
| **3. I went/would go to the hospital for re-examination regularly to adjust the medication when taking glucocorticoids.** | 123(30.98) | 121(30.48) | 65(16.37) | 25(6.30) | 63(15.87) |
| **4. I paid/would pay attention to the side effects of glucocorticoids when taking them.** | 108(27.20) | 91(22.92) | 85(21.41) | 37(9.32) | 76(19.14) |
| **5. I followed/would follow the doctor's instructions for home oxygen therapy.** | 128(32.24) | 67(16.88) | 50(12.59) | 24(6.05) | 128(32.24) |
| **6. I followed/would follow the doctor's instructions for pulmonary rehabilitation exercises.** | 60(15.11) | 69(17.38) | 58(14.61) | 42(10.58) | 168(42.32) |
| **7. I have quit/would quit smoking and have limited/would limit alcohol to prevent upper respiratory infections.** | 215(54.16) | 92(23.17) | 34(8.56) | 15(3.78) | 41(10.33) |
| **8. I ate/would eat more food high in vitamins, protein, and crude fiber, and eat less food high in animal fat and cholesterol.** | 128(32.24) | 149(37.53) | 80(20.15) | 23(5.79) | 17(4.28) |
| **9. I maintained/would maintain a healthy and optimistic mood to face the disease.** | 148(37.28) | 146(36.78) | 84(21.16) | 14(3.53) | 5(1.26) |
